# Effect of titanium oxide/reduced graphene (TiO_2_/rGO) addition onto water flux and reverse salt diffusion thin-film nanocomposite forward osmosis membranes

**DOI:** 10.1007/s11356-024-32500-0

**Published:** 2024-03-06

**Authors:** Amira M. Shawky, Yousra H. Kotp, Mahmoud A. Mousa, Mostafa M. S. Aboelfadl, Eisa E. Hekal, Khaled Zakaria

**Affiliations:** 1 Sanitary and Environmental Institute (SEI), Housing and Building National Research Center (HBRC), Giza, 1770 Egypt; 2 Hydrogeochemistry Dept, Desert Research Center, El Mataryia, Cairo, 11753 Egypt; 3 Chemistry Department, Faculty of Science, Benha University, Benha, Egypt; 4 Department of Chemistry, Faculty of Science, Ain Shams University, Abbassia, Cairo, 11566 Egypt; 5 Department of Analysis and Evaluation, Egyptian Petroleum Research Institute, Nasr City, 11727 Cairo Egypt

**Keywords:** Reduced graphene, Titanium dioxide, Oxide, Desalination, Thin-film composite membrane, Forward osmosis

## Abstract

Thin-film nanocomposite (TFN) forward osmosis (FO) membranes have attracted significant attention due to their potential for solving global water scarcity problems. In this study, we investigate the impact of titanium oxide (TiO_2_) and titanium oxide/reduced graphene (TiO_2_/rGO) additions on the performance of TFN-FO membranes, specifically focusing on water flux and reverse salt diffusion. Membranes with varying concentrations of TiO_2_ and TiO_2_/rGO were fabricated as interfacial polymerizing M-phenylenediamine (MPD) and benzenetricarbonyl tricholoride (TMC) monomers with TiO_2_ and its reduced graphene composites (TiO_2_/rGO). The TMC solution was supplemented with TiO_2_ and its reduced graphene composites (TiO_2_/rGO) to enhance FO performance and reverse solute flux. All MPD/TMC polyamide membranes are characterized using various techniques such as scanning electron microscopy (SEM), atomic force microscopy (AFM), and contact angle measurements. The results demonstrate that incorporating TiO_2_/rGO into the membrane thin layer improves water flux and reduces reverse salt diffusion. In contrast to the TFC membrane (10.24 L m^−2^h^−1^ and 6.53 g/m^2^ h), higher water flux and higher reverse solute flux were detected in the case of TiO_2_and TiO_2_/rGO-merged TFC skin membranes (18.81 and 24.52 L m^−2^h^−1^ and 2.74 and 2.15 g/m^2^ h, respectively). The effects of TiO_2_ and TiO_2_/rGO stacking on the skin membrane and the performance of TiO_2_ and TiO_2_/rGO skin membranes have been thoroughly studied. Additionally, being investigated is the impact of draw solution concentration.

**Graphical Abstract:**

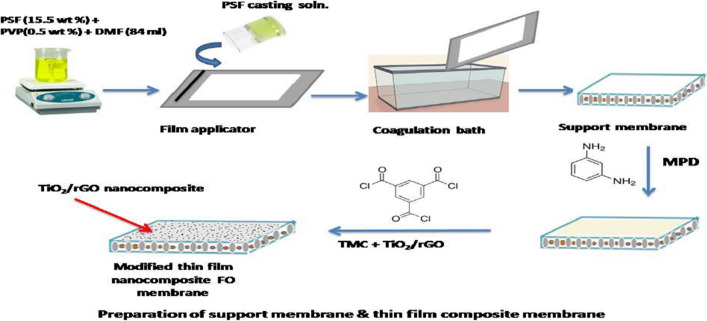

## Introduction

The best ways for introducing freshwater include desalination technologies including RO (reverse osmosis), MSF (thermal multi-stage filtration), and MED (multi-effect distillation). However, these processes are inefficient and energy-intensive, resulting in high water production costs (Elsaid et al. 2020; Curto et al. 2021). Forward osmosis (FO) is a method for water purification that involves semipermeable membranes for separating water from dissolved solutes, varying according to the purpose of application. The fundamental idea behind the FO process is based on the osmosis process that occurs naturally when water molecules move through a membrane from one region with a lower level of solute to another with a higher level. A draw solution with an elevated concentration of solutes is used to induce the osmotic pressure changes between both sides of the membrane in forward osmosis (Xu et al. 2017; Singh et al. 2021b). The resulting pressure variations cause water to flow from the feeding solution (which may include pollutants or impurities) towards the draw solution, effectively filtering the water. Here is a simple overview of the forward osmosis process (Chung et al. 2012, 2015): (1) feed solution: This is the water source that needs to be purified. It could be seawater, wastewater, or any other water source with impurities.

The best ways to introduce freshwater include desalination technologies, including RO (reverse osmosis), MSF (thermal multi-stage filtration), and MED (multi-effect distillation). However, these processes are inefficient and energy-intensive, resulting in high water production costs (Elsaid et al. 2020; Curto et al. 2021). Forward osmosis (FO) is a method for water purification that involves semipermeable membranes for separating water from dissolved solutes, varying according to the purpose of application. The fundamental idea behind the FO process is based on the osmosis process that occurs naturally when water molecules move through a membrane from one region with a lower level of solute to another with a higher level. A draw solution with an elevated concentration of solutes is used to induce the osmotic pressure changes between both sides of the membrane in forward osmosis (Xu et al. 2017). The resulting pressure variations cause water to flow from the feeding solution (which may include pollutants or impurities) towards the draw solution, effectively filtering the water. Here is a simple overview of the forward osmosis process (Chung et al. 2012, 2015):

1. Feed solution: This is the water source that needs to be purified. It could be seawater, wastewater, or any other water source with impurities.
2. Draw a solution: A solution with high levels of solutes is employed to produce an osmotic pressure change through the membrane.
3. Semipermeable membrane: a thin, porous material allowing water molecules to flow through in addition to restricting solutes and impurities from passing through.
4. Water purification: As water circulates between the feeding solution and the drawing solution, contaminants are left behind, resulting in purified water.
5. Draw solution recovery: The purified water in the draw solution is separated from the solutes, typically through methods like reverse osmosis, distillation, or membrane filtration.
6. Recirculation: The purified water is collected, and the draw solution is reused for continuous water treatment. Forward osmosis offers several advantages, including better energy consumption behavior, reduced fouling damage, and the ability to treat challenging water sources (Shawky et al. 2020). However, there are a number of limitations to the process, such as the requirement for an appropriate drawing solution and the extra process of recovering water out of the drawing solution. Forward osmosis has potential applications in a wide range of sectors, including food processing, pharmaceutical production, desalination, water treatment, and disposal. Forward osmosis (FO) membranes are semipermeable membranes that permit water to pass through while retaining solutes. FO membranes are classified into two distinct categories: cellulose-based membranes and thin-film composite (TFC) membranes (Suwaileh et al. 2018, Xu et al. 2019). Cellulose-based membranes, including both cellulose acetate and regenerated cellulose, were the first membranes used in FO. The aforementioned membranes possess outstanding water mobility but low salt rejection as compared to TFC membranes. They are also more susceptible to fouling, which can reduce their performance over time (Nguyen et al. 2013, Jin et al. 2016, Sharma et al. 2019).

TFC membranes, on the other hand, are made up of a thinner polyamide layer over a porous supporting layer. When compared to cellulose-based membranes, they exhibit more significant salt rejection and are more resistant to fouling. TFC membranes also have higher mechanical strength and chemical stability, making them more durable and appropriate for an expanded range of applications (Wei et al. 2011; Ren and McCutcheon 2014). In order to improve the performance of thin-film composite (TFC) skin membranes for forward osmosis (FO), inorganic nanoparticles (NPs) were introduced recently. The selectivity and water permeability, along with fouling resistance, of TFC membranes may all be considerably improved by these metal-based NPs (such as silver or copper), metal oxides (such as titanium dioxide or zinc oxide), or other metal types (Niksefat et al. 2014). The improved performance of TFC membranes incorporated with inorganic nanoparticles can be attributed to several factors:

- Increased hydrophilicity: Incorporating hydrophilic NPs into the TFC membrane can increase its water affinity, leading to an enhanced water flux. For example, adding titanium dioxide (TiO_2_) nanoparticles (Azad et al. 2022; Jain et al. 2022) to the TFC membrane has been shown to increase its hydrophilicity, resulting in a higher water permeability (Asempour et al. 2018).
- Improved mechanical strength: The membrane’s mechanical strength could also be improved through the loading of inorganic NPs, resulting in it being more resistant to compaction and deformation under high-pressure conditions. This can help maintain the membrane’s performance over extended periods (Pal et al. 2015, Singh et al. 2023).
- Reduced fouling: Incorporating inorganic NPs might reduce fouling and expand membrane lifetime by decreasing excessive foulant development over the membrane, the outer layer. The nanoparticles can create a more uniform surface, which reduces the adhesion of foulants, and they can also provide antimicrobial properties that restrict germs on the membrane’s surface from growing.
- Enhanced selectivity: Some types of inorganic NPs can improve the membrane’s selectivity by providing additional size-exclusion pathways or increasing the negative surface charge, which helps repel negatively charged solutes. This can improve a membrane’s capacity for rejecting undesired pollutants and enabling water to flow across it.
- Synergistic effects: In some cases, combining different types of inorganic NPs can lead to synergistic effects that result in even better FO performance. For example, in the combination of TiO_2_ and silver (Ag) nanoparticles (Chen et al. 2020a, Chen et al. 2020b), a TFC membrane has been shown to provide both improved hydrophilicity and antimicrobial properties. While the loading of inorganic nanoparticles in TFC skin membranes demonstrates great promise in terms of improving FO performance, further investigations are needed to optimize the fabrication processes, understand the long-term stability of these modified membranes, and investigate the potential environmental and health impacts of nanoparticle release. This research aimed to form the TiO_2_ layer and the embedded TiO_2_/rGO PA layer, as well as their basic composition, geometry, and properties. The effects of TiO_2_ and TiO_2_ and the effects of rGO loading on the membrane’s inherent separation efficiency, morphology, FO, and antifouling efficacy were thoroughly investigated.

## Experimental

### Chemicals

Polyvinylpyrrolidone (PVP K30 from Sigma-Aldrich) was utilized as an additive, hexane solvent (advent, > 99%, HPLC grade), and a membrane substrate, polysulfone (Udel P-3500 in pellet), supplied by Solvay Advanced Polymers. 1,3,5-Benzenetricarbonyl trichloride (TMC, > 98%) as well as 1,3-phenylenediamine (MPD, > 99%) were supplied from MERK Chemical Company. Graphite (99.995% purity) is supplied from Fluka, Switzerland. Potassium permanganate 97% (KMnO_4_), hydrazine monohydrate 99% (NH_2_-NH_2_.H_2_O), sodium nitrate 95% (NaNO_3_), hydrogen peroxide 30% (H_2_O_2_), ethanol 99%, (C_2_H_5_OH) sodium sulfate 98% (Na_2_SO_4_), sulfuric acid 98% (H_2_SO_4_), and ammonia solution (28 wt%) were obtained from Adwic Pharmaceutical and Chemical Company in Egypt, and titanium (IV) isopropoxide 98% (C_12_H_28_O_4_Ti) was purchased from Acros Chemical, NJ, USA. None of the commercially available, analytical-grade compounds needed further purification before use.

### Synthesis methods

#### Preparation of titanium dioxide (TiO_2_)

Using the sol–gel technique, titanium isopropoxide was dissolved in an appropriate solvent, for example, water or ethanol, to generate a clear solution of TiO_2_. The obtained sol solution was regularly stirred at room temperature for a period of 4 h in a glass beaker in order to generate TiO_2_ powder. After filtering off the precipitated powder, it was cooked up in a furnace at 60 °C overnight. The produced powder material was further calcined at 500 °C for 1 h. In the end, the TiO_2_ particles were thoroughly ground in a mortar (Sharma et al. 2014).

#### Preparation of titanium dioxide nanocomposite with reduced graphene (TiO_2_/rGO)

One gram of the reduced graphene oxide, 0.5 g of the acetyl trimethylammonium bromide (CTAB), and 30 ml of ethanol as a solvent were mixed simultaneously in a 100-ml beaker under stirring. Eleven milliliter of titanium (IV) isopropoxide was injected into the reactor one drop at a time after the initial period of 30 min. Following that, 20 ml of distilled water was further poured into the mixed solution. After continuous stirring of the suspension for about 8 h, it was cooled to 80 °C. The resultant material was annealed in a muffle furnace for 5 min at 500 °C before being gently cooled to the ambient temperature (Atout et al. 2017).

#### Fabrication of a support membrane using polysulfone (PSF)

The PSF substrate was generated via the phase-inversion process. Just before being properly mixed at 700 rpm at 70 °C, PSf (15.5 wt%) and PVP (0.5 wt%) beads were totally added to the DMF solution. To remove any gases that may have become trapped, stir the polymer solution for 12 h at room temperature. A 100-micron polyester fabric veil is set over a crystal-clear glass disc. The resulted dope solution was subsequently applied to a non-woven polyester via a TQC automated film applicator with a film thickness of about 50 µm. The glass slide was gently inserted into distilled water (a coagulation solution) at ambient temperatures in order to start the phase separation operation. To get rid of any solvent along with other impurities, the final substrate was submerged in distilled water for about 24 h. Before proceeding to the subsequent section, the substrate material was sandwiched between two sheets of filtration paper (Emadzadeh et al. 2014b).

#### Developing a thin-film hybrid membrane

Interfacial polymerization technique was applied to generate the active layer on a manually cast PSf framework. The PSf support was submerged in a solution of MPD (2.0 wt%) with 0.15 wt% SLS around 2 min to generate a TFC polyimide membrane structure. The remaining MPD solution was subsequently scraped off the outer layer using rubber rollers. Following that, 0.53 wt% TMC hexane solution was collected from the surface after 1 min of contact. After that, the TFC-FO membrane was left to cure in a hot oven at 80 °C for 5 min. The technique was applied again after holding it in deionized water at approximately 40 °C for 5 min. The fabrication of TFN-TiO_2_/rGO and TFN-TiO_2_ membranes was identical to that of TFC membranes, except for the preloading with the nanomaterials from a 0.53 wt% TMC solution. A TMC n-hexane solution was ultrasonically dispersed 1 h using different nanomaterial ratios (0.3, 0.5, 0.7, and 0.9% by weight), interfacial polymerization with the resultant solution and the MPD-wetted PSF support produced TFN-TiO_2_ and TFN-TiO_2_/rGO films (Emadzadeh et al. 2014a).

### Characterization

A multi-technique approach, including X-ray analysis, spectroscopy, profilometry, and microscopy, as well as measurement of contact angles, was employed for assessing the membranes and nanomaterials in terms of morphology, crystallinity, roughness, composition, and wettability. According to the description, the membranes with nanomaterials were defined using the following techniques:

1. Diffractometry with X-rays: Used to assess material crystallinity along with phase composition. At ambient temperature, a Philips X’Pert Pro Super diffractometer and Cu Kα radiation were used. Diffraction patterns were detected by scanning from 5 to 80° in 2θ directions.
2. Raman spectroscopy: In order to assess the Raman spectra of the materials, a U-1000 laser Raman spectrometer with a 514.5 nm excitation wavelength was employed. It provides details about a material’s rotational, vibrational, and other low-frequency modes.
3. Transmission electron microscopy (TEM): High-resolution TEM images and morphology at the nanoscale were captured using an FEI Tecnai G20 TEM at 200 kV. Before imaging, specimens of powder were set on carbon-coated copper grids. To preserve the sample structure, pictures were taken with little electron irradiation.
4. Fourier transform infrared spectroscopy (attenuated total reflectance) (ATR-FTIR): A JASCO FT-IR 6100 spectrometer was employed for assessing the existence of nano-carbon elements, particularly in the polyamide layer. The IR absorption spectrum is used for identifying chemical bonds and functional groups.
5. Scanning electron microscopy (SEM): A Quanta FEG250 SEM at 30 kV was used to analyze the surface morphologies of the films at increasing magnifications. 3D surface photos with nanoscale resolution are demonstrated.
6. Atomic force microscopy (AFM) was utilized to investigate the surface roughness and texture of membranes at the nanoscale through an AFM Flex Nano Surf C3000 in static contact mode. The average surface roughness was identified by scanning 2.5 μm × 2.5 μm regions at various places.
7. A VCA Video Contact Angle System had been used to measure the static water contact angles of membranes at ambient temperature. Surface hydrophobicity as well as hydrophilicity are evaluated. At least six measurements were conducted at various points on each membrane sample.

### Measuring membrane intrinsic separation properties

The membrane’s salt rejection (*R*), net water permeability (*A*), and NaCl rejection (*B*) were all evaluated in a cross-flow reverse osmosis (RO) system. 42 cm^2^ was utilized by the membrane. While the transverse flux is substantial (0.32 m/s), the lattice dielectric lowers the concentration of external polarization. Permeability is calculated using the net drain flow and the transmembrane pressure, which is 4 bar (Arjmandi et al. 2020):

1 $$A =\frac{{\varvec{J}}}{\Delta {\varvec{P}}}$$

where Δ*P* denotes the trans pressure of the membrane and *J* denotes the flux of water permeable. By determining the salinity of both the feeding and permeates, the salt rejection (*R*) could be estimated (Arjmandi et al. 2020):

2 $$R = 1-\frac{{{\varvec{C}}}_{{\varvec{P}}}}{{{\varvec{C}}}_{{\varvec{F}}}}$$

The salt level concentrations in the feeding water as well as permeate, respectively, are represented by the characters *C*_*f*_ and *C*_*p*_. The intrinsic separation properties of membranes controlled the salt permeability of the membrane.

3 $$B =\frac{1}{R}-1$$

### Performance measurement of the forward osmosis (FO)

The efficiency of FO the process (salt and water flow) was estimated using benchscale FO equipment, which is demonstrated by Shawky et al. (2020). The feeding and drawing solution velocities were kept constant at 1.6 L/h for the FO tests. Furthermore, the feeding and drawing solutions’ temperatures were kept fixed at 23 ± 2 °C. Both solutions employed for drawing and feeding were 1 M and 10 mM NaCl, respectively. Furthermore, investigations have been conducted on the membrane orientations AL-DS and AL-FS. The flow rate of water permeation was estimated using a computerized mass balance using the weight difference between the feeding and drawing streams. For calculating the FO water flow, the total quantity of water that was transferred from the pump’s feeding side to the drawing side (*V*) was determined and included in the formula below (*J*_*v*_) (Shakeri et al. 2019):

4 $${J}_{v}=\frac{\Delta V}{\Delta t\times {A}_{m}}$$

The area of the membrane’s surface is expressed by *A*_*m*_, while the time variation is expressed by Δ*t*. In the opposite direction of water penetration, reverse salt flow (flux) (*J*_*s*_) develops from the drawing side to the feeding side. By dividing the membrane area by the mass flow rate of NaCl, the reverse salt flux was calculated (Shakeri et al. 2019).

5 $${J}_{s}=\frac{{V}_{t}{C}_{t }- {V}_{0}{C}_{0}}{\Delta t \times {A}_{m}}$$

*C*_*o*_ and *C*_*t*_ stand for the starting and ending salt levels, respectively, along with *V*_*o*_ and *V*_*t*_ for the starting and ending feeding solution volumes. The trials were held at 23 ± 2 °C.

The formula (6) that is listed below describes the salt rejection, *R*, in the FO process:

6 $$R=\frac{(1- {J}_{s}/{J}_{V})}{{(C}_{d 0}+ {C}_{ d e}/2)} \times 100$$

*J*_*s*_ (g/m^2^.h) represents the reverse solute flux, *J*_*w*_ (L/m^2^.h) represents the water flowing through FO, and *C*_*d* 0_ and *C*_*d e*_ are the starting as well as final concentrations of the solution used for draw (gm/L), respectively.

## Results and discussion

### TiO_2_ and TiO_2_/rGO nanomaterial characterization

**Fig. 1:**
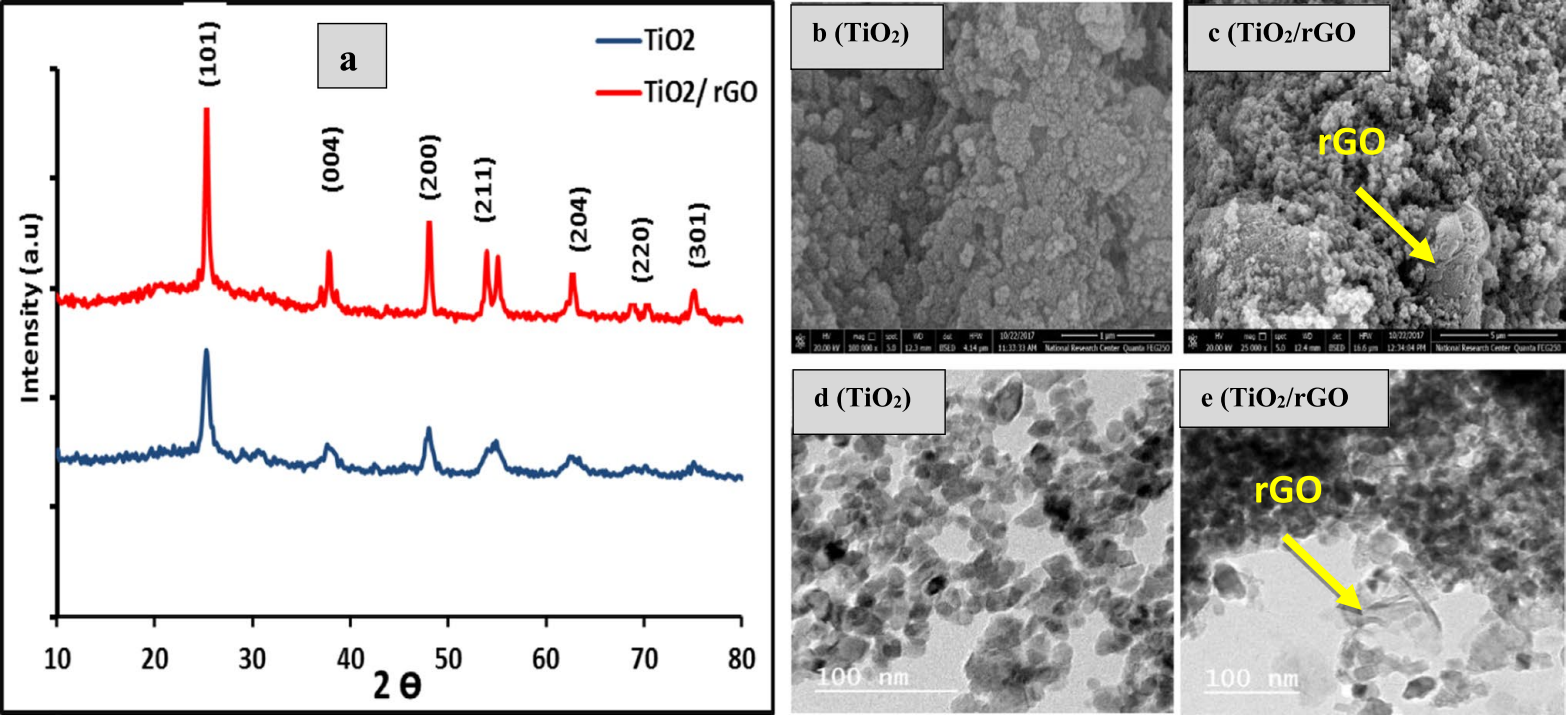
**a** XRD patterns of the prepared TiO_2_ and TiO_2_/rGO samples, **b**, **c** SEM images of TiO_2_ and TiO_2_/rGO samples, and **d**, **e** TEM of nanomaterials TiO_2_ and TiO_2_/rGO

Figure 1a illustrates the patterns generated by XRD of as-synthesized TiO_2_ and TiO_2_/rGO samples. According to the diffraction peaks at 25.33°, 38.03°, 48.02°, and 53.89°, which correspond to lattice planes (101), (004), (200), (105), and (333), singly with the structure of anatase matched with JCPDS card No. 84–1286, on the sample preparation, the anatase phase was completely organized (Sharma et al. 2014).

**Fig. 2:**
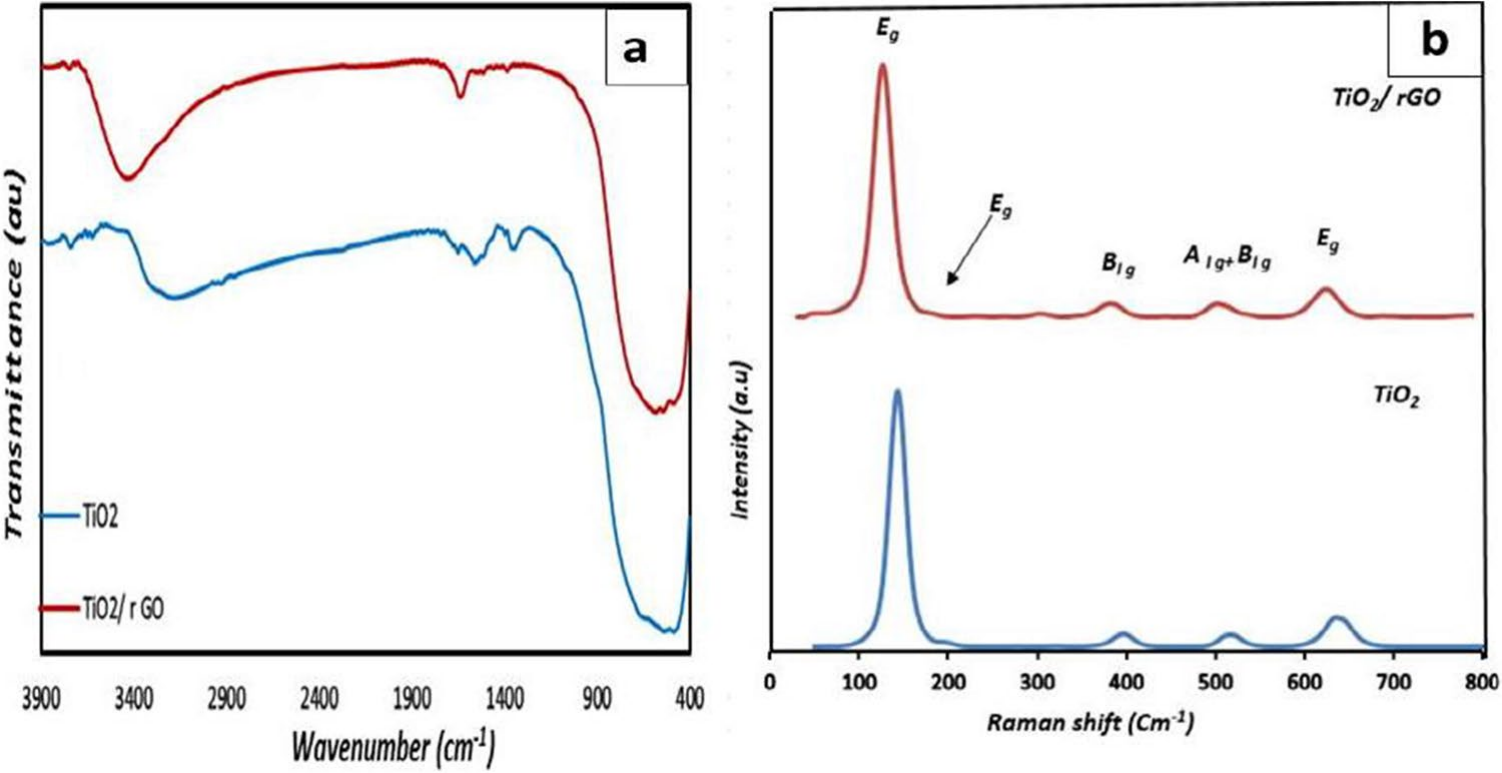
**a** FTIR spectrum of the prepared TiO_2_ and TiO_2_/rGO samples. **b** Raman spectra of the prepared TiO_2_ and TiO_2_/rGO samples

While reducing graphene composite with TiO_2_, there are no peaks except anatase. Besides, the diffraction peak of rGO at ~ 26° was possibly stacked with the leading peak of TiO_2_ (Atout et al. 2017). Raman vibrations were visible in the anatase TiO_2_ at approximately 146, 199, 400, 519, and 636 cm^−1^. No rutile peaks were seen, and all peaks match the anatase crystalline structure. Ti–O stretching mode peaks are detected at 639 cm^−1^ (*E*_*g*_), 517 cm^−1^ (*A*_1*g*_ + *B*_1*g*_), and 397 cm^−1^ (*E*_*g*_). The O-Ti–O bending mode’s peaks are located at 397 cm^−1^ (*E*_*g*_) (Liang et al. 2014). The amount of reduced graphene in TiO_2_ with reduced graphene (TiO_2_/rGO) is minimal; therefore, the graphene peak does not emerge, although a slight peak shift was found when comparing anatase TiO_2_. Because the amount of rGO is so small, it does not appear in Raman diffraction, as seen in Fig. 2b (Luo et al. 2015). All of the studied samples’ FT-IR spectra are shown in Fig. 2a, which demonstrates a prominent absorbance band that emerged in the 400–700 cm^−1^ region. This band is associated with the formation of TiO_2_ and the stretching vibration of the Ti–O-Ti bond. The apparent shift in this band is consistent with the dopants’ incorporation into the TiO_2_ lattice. It was also noted that the O–H bending vibration occurred in a separate vibration band with dimensions of 1622 to 1796 cm^−1^. This could be explained by the existence of H_2_O molecules that have been adsorbed on TiO_2_. Adsorbed H_2_O molecules can be attributed to the vibration band seen at around 3400 cm^−1^. The TiO_2_/rGO showed several absorbance peaks higher than those noticed in the FT-IR spectrum of pure TiO_2_ revealing the construction of new species during the doping process (Heo et al. 2019). The morphology of TiO_2_ and TiO_2_/rGO nanomaterials measured by SEM is given in Fig. 1b, c. Round, sponge-like aggregates are formed by undoped anatase grains. Reduced graphene sheets appear slightly due to the condensation and accumulation of titanium oxide particles on the surface of the graphene, which helps to cover them completely (Pugazhenthiran et al. 2020). The TEM images of TiO_2_&TiO_2_/rGO show the spherical shape with different particle sizes in Fig. 1d, e. According to the TiO_2_ microscopy, the nominal size of the nanoparticles was around 9 nm, and they looked to be moderately agglomerated yet rather homogeneous and uniform in size. In the case of TiO_2_ with reduced graphene, the reduced graphene flakes appear stacked on their surface with titanium oxide (Azani et al. 2019).

### Characterization of membranes (TFC, TFN-TiO_2_, and TFN-TiO_2_/rGO)

**Fig. 3:**
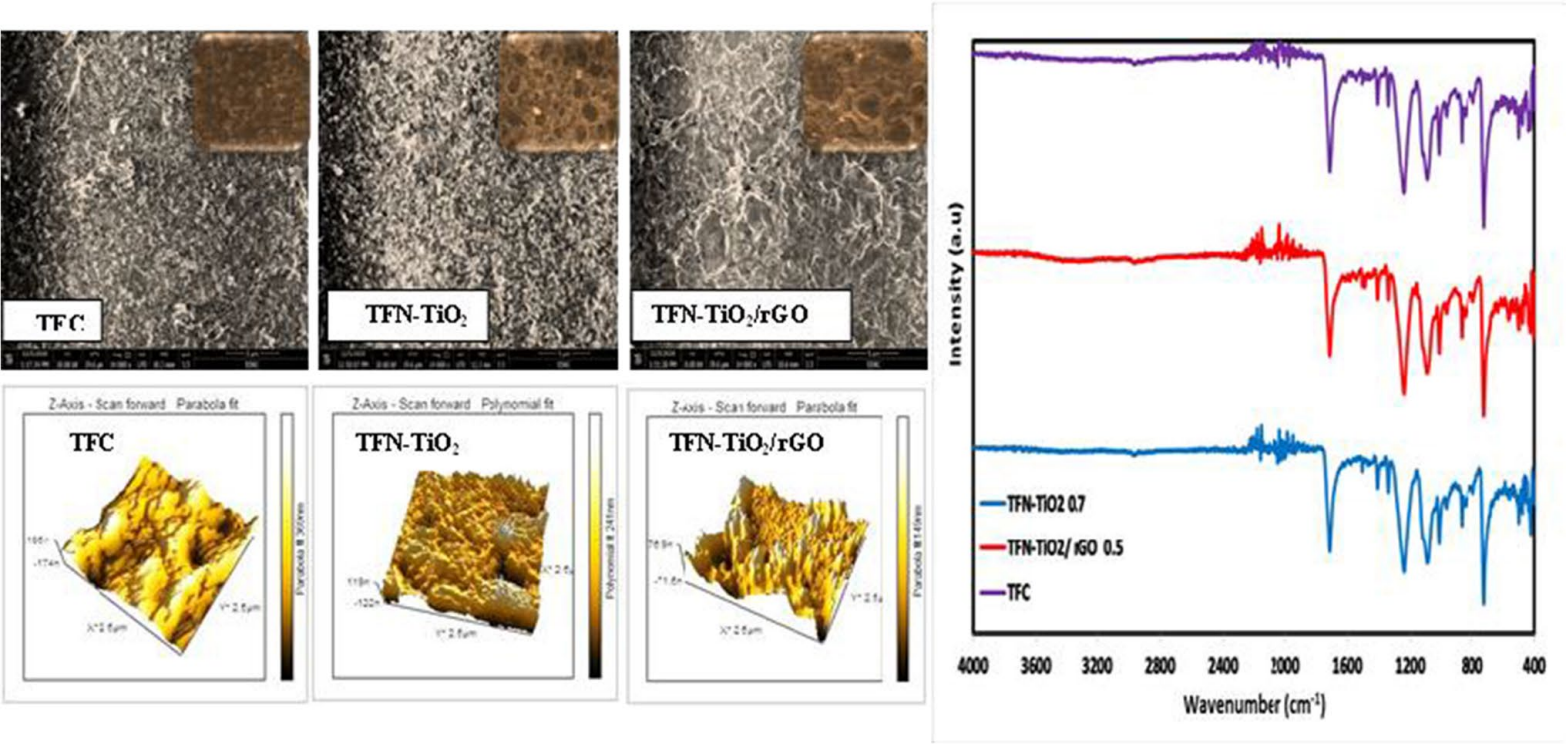
ATR-FTIR of TFC, TFN-TiO_2_, and TFN-TiO_2_/rGO; SEM of TFC, TFN-TiO_2_, and TFN-TiO_2_/rGO; and AFM of TFC, TFN-TiO_2_, and TFN-TiO_2_/rGO

The ATR-FTIR spectra corresponding to each papered membrane, including TFC, TFN-TiO_2_, and TFN-TiO_2_/rGO, are represented in Fig. 3. TFC and TFNs exhibit absorption bands at 1654 cm^−1^ and 1544 cm^−1^, demonstrating that the PA layer is developing effectively. These absorption bands are correlated with both amine (N–H) bending vibrations of the amide groups in addition to carbonyl (C = O) stretching (Emadzadeh et al. 2014a; Bagherzadeh et al. 2020). When compared against the TFC membrane, the peak broadening of the TFN-TiO_2_/rGO and TFN-TiO_2_ membranes at 1544 cm^−1^ demonstrates the emergence of an extra bond (C-O) between the TiO_2_ and the unbound -COCl of TMC. Since some of the -COCl units within TMC do not interact via interfacial polymerization with MPD, the remaining -COCl units would react with the groups of OH in TiO_2_ (Akther et al. 2019), as illustrated in Fig. 3. Each of the TFC, TFN-TiO_2_, and TFN-TiO_2_/rGO membrane surfaces will be observed in top-view images obtained from SEM of the membrane, which further illustrate their different surface morphologies. The TFC, TFN˗TiO_2_, and TFN˗TiO_2_/rGO membranes showed crest structures on their surfaces. Figure 3 displays a polyamide (PA) membrane characteristic fabricated via the interfacial polymerization technique. With regard to the denser structure of TFN membranes, careful study reveals that the TFC membrane displayed a more nodular surface. This demonstrates how the incorporation of nanomaterials (TiO_2_ and TiO_2_/rGO) caused significant changes in membrane surface structure. The ridge-valley structure of TFN membranes is ascending and enlarged. This is because during the interfacial polymerization process, carboxyl and TMC react at a slower rate than MPD and TMC. Additionally, the hydroxyl groups on the nanomaterials produce hydrogen bonds that make the molecular chains more compact. The interfacial polymerization process’s pace and hydrogen banding’s impact on shaping the surface of the membrane were both influenced by the level of thickness of the polyamide film adhered to the surface of the substrate (Li et al. 2019a). The 3D AFM photographs of both TFC and TFN skin membranes are shown in Fig. 3. The “ridge-and-valley” PA configuration is dispersed out all over the plane within the TFC together with TFN membranes, as can be seen from the AFM images. AFM studies show that the deep depressions (pores) and nodules on the surface were improved by the presence of nanomaterials. Additionally, the height of the aggregates on the surface is linked to the substrate’s roughness (Li et al. 2020; Zhang et al. 2020). By increasing the amount of nanomaterials (TiO_2_ and TiO_2_/rGO) in the TMC solution, the surface roughness increased.

According to the R_MS_ values of TFC and TFN membranes with equal support layers, the surface roughness is increased when nanomaterials are present. The R_MS_ value of TFN˗TiO_2_/r GO, for instance, is larger than the R_MS_ value of the TFC membrane, which is 15.3 nm. The root mean square of the *Z* value, R_MS_, the mean height difference between the peaks of the TFC sample and the eventual TFN, and the average surface roughness (R_a_) are found to have changed. For easier comparison, Table 1 includes a statistical list of these shifts in the standard parameters. Surface hydrophilicity is one of the most important characteristics that could significantly impact the flux and antifouling capacity of membranes. Table 1 lists the values of the contact angle for the TFC and TFN membranes.

**Table 1:** AFM data and contact angle measurements of synthesized TFC, TFN-TiO_2_, and TFN-TiO_2_/rGO membranes

Membrane	*R*_*a*_ (nm)	*R*_*Ms*_ (nm)	*R*_*z*_ (nm)	Contact angle θ (°)
TFC	10.58	15.34	212.07	64.8
TFN-TiO_2_ 0.7	29.36	38.01	308.74	59.2
TFN-TiO_2_/rGO 0.5	37.54	47.61	362.51	54.7

A lower number indicates better hydrophilicity and a higher water propensity, whereas the contact angle value illustrates the membrane surface’s propensity to soak water. The degree of hydrophilicity is inversely correlated with the degree of surface WCA. The membrane surface hydrophilicity is increased by the hydrophilic functional groups of nonmaterial (i.e., carboxyl and hydroxyl). Another factor contributing to the improvement of the WCA is the greater surface roughness of the membranes caused by the loading of varying amounts of nonmaterial (Li et al. 2020).

## Membrane separation properties

### Impact of stacking of TiO_2_ and TiO_2_/rGO on the separation properties of manufactured membranes

A cross-flow RO experimental setup was used to determine the impact on NaCl rejection and water flux when loading the membrane with TiO_2_ and TiO_2_/rGO. The results are shown in Table 2.

**Table 2:** Separation properties of synthesized TFC, TFN-TiO_2_, TFN-TiO_2_/N, and TFN-TiO_2_/rGO FO membranes

FO membrane	Pure water permeability, A (L/(m^2^h bar))^a^	Salt rejection, R (%)	Salt permeability, B (L/m^2^h)^b^	B/A
TFC	1.72	89.2	0.21	0.12
TFN-TiO2 0.7	3.27	95.7	0.14	0.04
TFN-TiO2/rGO 0.5	3.63	96.9	0.12	0.03

^a^DI water is used as the feed solution in the RO test with an applied pressure of 4 bar (2.5 L/min)

^b^2000 ppm NaCl solution is used as the feed solution in the RO test with an applied pressure of 4 bar (2.5 L/min)

**Fig. 4:**
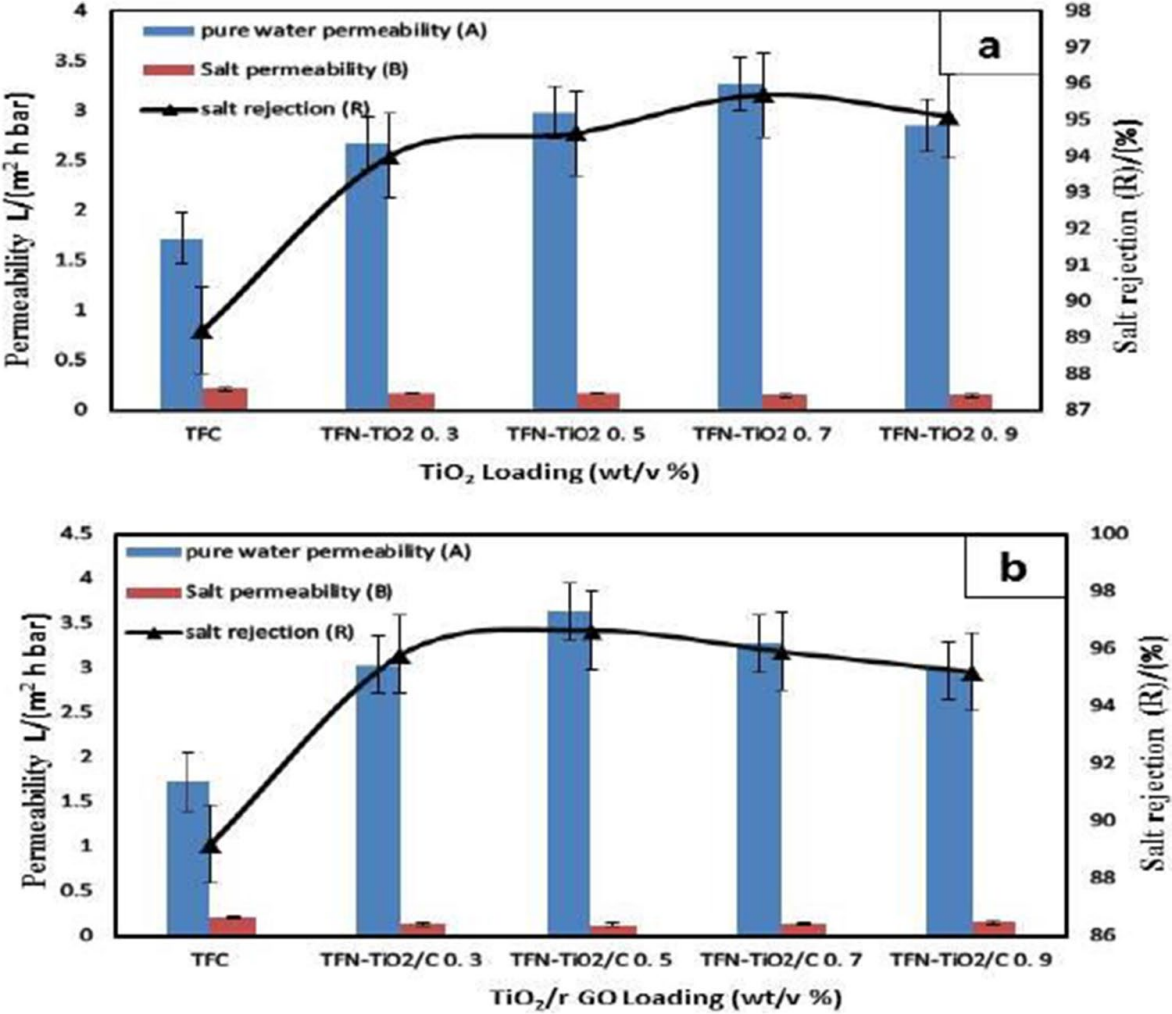
Water permeability and salt permeability of synthesized **a** TiO_2_-FO membranes and **b** TiO_2_/rGO-FO membranes

According to Fig. 4a, A=3.72 L/m^2^ h bar for TFN-TiO_2_ 0.7 vs. 1.73 L/m^2^.h.bar for TFC, the incorporation of TiO_2_ increased the water permeability. The improved surface texture, such as the valley, ridge, and leaf-like structure after the addition of TiO_2_, and the increased membrane hydrophilicity are both responsible for some of this flow augmentation (reduced water contact angle). The water permeability decreases with increasing TiO_2_ content (0.9%), nevertheless. TiO_2_ may aggregate at higher concentrations, which would explain the thin-film layer’s non-uniform distribution of TiO_2_ (Rajakumaran et al. 2019).

In comparison to the TFC membrane, more salt was rejected by the TFN membranes. When 0.7% TiO_2_ was added, the TFC membrane’s salt rejection increased from 89 to 95.7%. Because of this, the salt rejection rises to 0.7% before falling due to the selective layer’s TiO_2_ loading of 0.9%. If there is hydrophilic TiO_2_ in the organic phase, the aqueous and organic phases might mix more easily during the interfacial polymerization process. However, excessive nanoparticle loading reduced the degree of cross-linking and increased the number of microvoids between the nanofillers and the polymer matrix. Lowering salt rejection might improve water diffusion.

Much higher water permeability was demonstrated by each TFN membrane than the TFC control membrane. Since the TFN-TiO_2_ 0.7 membrane had the lowest salt permeability/water permeability (B/A) ratio of all the membranes tested, it was found to be the most effective at reducing solute reverse diffusion throughout the FO process. The TFN-TiO_2_ 0.7 membrane appears to be the most practical for FO application due to its strong rejection, low B/A ratio, and moderately high water permeability (Li et al. 2019a; Wu et al. 2020).

However, the amount of water permeability rose from 1.05 to 4.6 L/m^2^ h as the TiO_2_/rGO dose rose from 0.0% to 0.5% w/v. The increase in water permeability in the TFN membrane accounts for almost all of the elevation in membrane hydrophilic characteristics. The improved hydrophilicity allows molecules of water to dissociate more quickly and flow through the membrane. The increased roughness of the membrane surface may possibly explain some of the increase in water flux. TFN membranes repelled salt more well than TFC membranes (0.3, 0.5, 0.7, and 0.9 wt% TiO_2_/rGO loading). The increase in salt rejection from 89% in the original membrane to 0.5 wt% TiO_2_/rGO in the modified membrane is due to TiO_2_/rGO nanoparticle pore blockage. Despite this, as illustrated in Fig. 4b, a slight reduction was observed at the maximum TiO_2_/rGO dosage (0.7% w/v). This is owing to the lower ion rejection rate of aggregated TiO_2_/rGO nanoparticles (Rajakumaran et al. 2019). The selectivity of a FO membrane is proportional to its B/A ratio. Table 2 also contains the B/A ratio data. Lower B/A proportion loading ratios in membranes are frequently employed in the FO approach due to their tendency to reduce reverse solute diffusion through the DS to the FS. In other words, enhancing the selectivity of the membrane could decrease the trend of the B/A proportion by elevating the water’s permeability coefficient and diminishing the salt’s permeation coefficient, the two parameters that have a direct relationship with the efficiency of the FO membrane (Li et al. 2019b; Saeedi-Jurkuyeh et al. 2020). The B/A ratio decreased in all membranes that were generated as the TiO_2_/rGO concentration rose.

## FO performance

### Effect of TiO_2_ and TiO_2_/rGO loading on FO performance

**Fig. 5:**
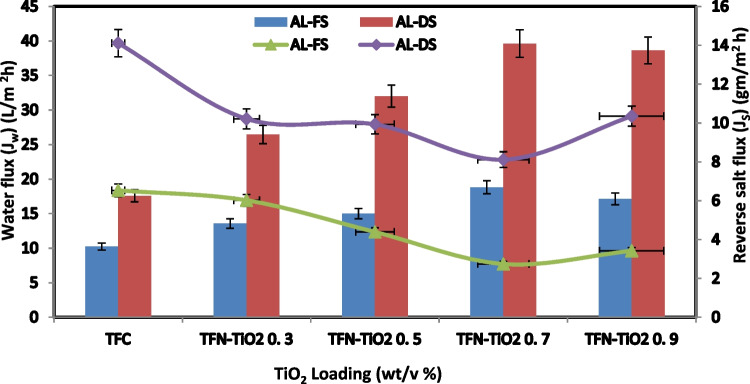
FO water flux and solute flux of synthesized TiO_2_-FO membranes

The water permeability of the developed TFC and TFN-FO membranes was found to be significant in the AL-DS (active layer extraction solution) and AL-FS (active layer feed solution) directions, where FS contained deionized water and DS contained 1 M NaCl. On the other hand, water flow increases from an AL-DS to an AL-FS direction. Figure 5 shows the results, which show that the modified membrane has a larger water and solute flux than the TFC membrane. The water flux in the TFC membrane rose considerably from AL-DS mode to 10.24–18.81 L/m in FO mode for TFN-TiO_2_0.7. Two factors may affect the increase in water flux caused by the addition of TiO_2_ from 0.3 to 0.7%: To make the TFN-FO membrane more hydrophilic and, thus, increase the water flux, TiO_2_ nanoparticles can be added. The packing of polymer chains is impacted by the mobilization of TiO_2_ nanoparticles. Contact angle measurement results can confirm this. It has been shown that asymmetric membrane orientation greatly affects how the membrane functions. As opposed to the higher AL-FS, the AL-DS had a lower internal concentration polarization, making it the optimum choice for flow optimization. Figure 5 shows the solute transport in both directions across the FO membranes. The solute flow through all produced membranes is increased by adding more TiO_2_ nanoparticles. The concentration of nanostructures may be a limiting factor in TFN-0.9’s enhanced solute flux when compared to other TFN membranes since this prevents polyamide coatings from forming thin films as effectively (Li et al. 2019b; Saeedi-Jurkuyeh et al. 2020).

**Fig. 6:**
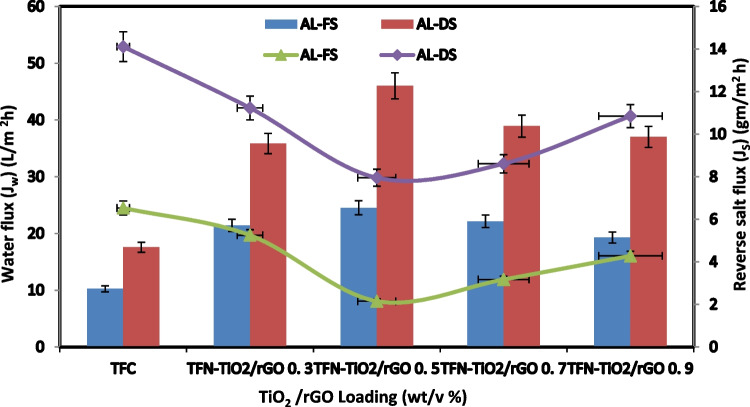
FO water flux and solute flux of synthesized TiO_2_/rGO-FO membranes

Figure 6 displays the functional groups that TiO_2_/rGO effectively loaded onto the FO membrane. Table 3 shows the membrane water flux for both AL˗FS and AL˗DS directions as calculated by means of the FO approach. With TFN˗TiO_2_/rGO 0.5 in AL˗DS mode and also from 10.24 to 24.52 L/m^2^ h in the FO approach, the TFC membrane water flux increased. The higher permeability of the TiO_2_/rGO/FO membrane is a result of TiO nanoparticles’ increased hydrophilicity, hydroxyl groups’ ability to hydrolyze them, and the abundance of hydrophilic functional groups on their surface (Emadzadeh et al. 2014b; Shawky et al. 2020). Due to the presence of reduced graphene, TiO_2_ was more equally distributed throughout the membrane’s surface. Reduced graphene sheets made the surface rougher, which increased its hydrophilicity and caused the water flux to raise the concentration of TiO_2_/rGO. Figure 6 shows the solute flux through membranes in both directions. TFN membranes have lower reverse salt fluxes overall than TFC membranes. The solute flux decreased as the TiO_2_/rGO 0.5 loading was increased. The reason that TFN 0.7 has a higher solute flux than other TFN membranes may be due to the accumulation of nanostructure, which hinders the development of an ideal polyamide thin film (Han et al. 2012). The ideal concentration of TiO_2_/rGO in the nanocomposite membrane’s active layer can be determined based on the water and solute fluxes. To make comparisons simpler, Table 3 now contains the *J*_*S*_/*J*_*W*_ ratio of the water flux to the reverse salt flux.

**Table 3:** Comparison of the FO membrane performance in the FO/PRO modes between the FO membranes developed in this study and the FO membranes reported in the literature

FO membrane	Water flux (*J*_*w*_) (L/m^2^ h)		Reverse salt flux (*J*_*s*_) (g/m^2^ h)		*J*_*s*_/*J*_*W*_ (g/L)		RFO	FS	DS (M NaCl)	Rfc
	AL-FS	AL-DS	AL-FS	AL-DS	AL-FS	AL-DS				
TFC	10.24	17.58	6.53	14.11	0.64	0.8	98.91	DI	1 M	This work
TFN-TiO_2_ 0.7	18.81	39.62	2.74	8.11	0.04	0.19	99.99	DI	1 M	This work
TFN-TiO_2_/rGO 0. 5	24.52	46.02	2.15	7.95	0.08	0.17	99.99	DI	1 M	This work
PA-GO	14.5	34.7	2.6	4.7	0.17	0.13	––	DI	1 M	[50]
PA-TiO_2_	17.1	30.2	2.9	6.66	––-	––-	––-	10 m M NaCl	0.5 M	[27]
PA-TiO_2_	29.7	56.7	7.3	14.4	––-	–––	––-	10 m M NaCl	2 M	[27]
TFN-UiO-66-2GO	25.52	–-	6.9	––	––	–––	–––	DI	2 M	[53]
PA-GQDs	12.9	––	1.41	––-	0.11	––-	––-	DI	0.5 M MgCl_2_	[52]
TFN-Fe_3_O_4_/ZnO	29.3	––-	5.9	––-	0.35	––-	–––	10 m M NaCl	2 M	[55]

### Membrane orientation’s effect on the FO process’s performance

According to studies, the orientation of the membrane can affect the rate of rejection of salt and water flow. In a conventional FO approach, the membrane is positioned so that the active membrane layer faces the solution that forms the feed. This arrangement enables a more substantial rejection of solutes and particles in the feeding solution, potentially improving the draw solution’s quality. This orientation, however, can lead to membrane fouling as the feeding solution can deposit particles and other impurities on the active layer. The membrane can alternatively be positioned such that the active layer is towards the drawing solution. Due to the draw solution’s lower flow resistance, this design can boost the amount of water that passes through the membrane. However, this orientation can also result in lower selectivity, as solutes in the feeding solution are more likely to pass through the membrane (Zhao et al. 2011; Wang et al. 2016). Table 3 demonstrates the efficacy of the FO technique by employing TFC and TFN filter membranes in response to membrane orientation. Water flow is significantly higher in AL-DS mode as compared to AL-FS mode in TFC and also in TFN membranes fed with water from DI containing 0.1 M NaCl. This is because the membrane support layer’s structural strength and porous characteristics impact water flow. Water enters the membrane in AL-FS mode from its feeding side, dilating the DS and reducing its osmotic pressure and the flow of water. High DS osmotic pressure is required for the FO process in order to drive water flow. But the overall water flux is higher than in AL-FS mode. Dilute ICP (internal concentration polarization) in AL using 0.1 M NaCl as DS further reduces water flux. In AL-DS mode, the concentrated DS on one side of the active layer reduces driving force and water flux slightly. DS mode also reduces water flux, but flux is still higher than AL-FS mode (Wei et al. 2021). Water flow is higher with DI water compared to 0.1 M NaCl feed in AL-FS and AL-DS modes. This is because concentrated ECP forms near the active layer with 0.1 M NaCl feed, reducing the driving force. Compared to FO mode, higher water flux is achieved in DS mode since the concentrated dissolved solids at the membrane surface provide a higher driving force. The DS mode is not recommended for desalination applications due to the feeding solution that raises the permeable support layer’s susceptibility to fouling (Jin et al. 2012; Shokrgozar Eslah et al. 2018). Reverse solute flowing (RSF) along with specific reverse solute flowing (SRSF) is also increased in DS mode due to greater DS concentration level and the driving force.

### The influence of draw and feed concentrations on TFC, TFN-TiO_2_, and TFN-TiO_2_/rGO membranes

The different concentrations of draw and feed affected the forward osmosis process in different directions:

1. Water flux: As the drawing concentration rises, the osmotic pressure variations increase, increasing the water flow through membranes. Water flux, on the other hand, tends to decrease when feed concentration increases due to increasing osmotic pressure on the feed side.
2. Reverse solute flux: An increase in draw concentration can result in a higher reverse solute flux, as the concentration gradient thought that the membrane becomes more significant. This frequently leads to more solute leakage through the membrane. Conversely, a higher feed concentration will result in a diminished reversed solute flux since the gradient of concentration will be decreased.
3. Membrane fouling: Higher feeding concentrations may lead more solute particles to settle on the outer surface of the membrane or enter the membrane surface’s pores, resulting in an increased membrane fouling rate. This can negatively affect the performance of membranes. Higher draw concentrations may contribute to fouling because the membrane itself will become more permeable to the drawing solution’s solute, which leads to fouling on the feeding side.
4. Separation efficiency: The separation efficiency of membranes is influenced by both draw and feed concentrations. Higher draw concentrations can improve the water flux and lead to increased separation efficiency, while higher feed concentrations can negatively impact the separation efficiency due to reduced water flux and increased fouling.

**Fig. 7:**
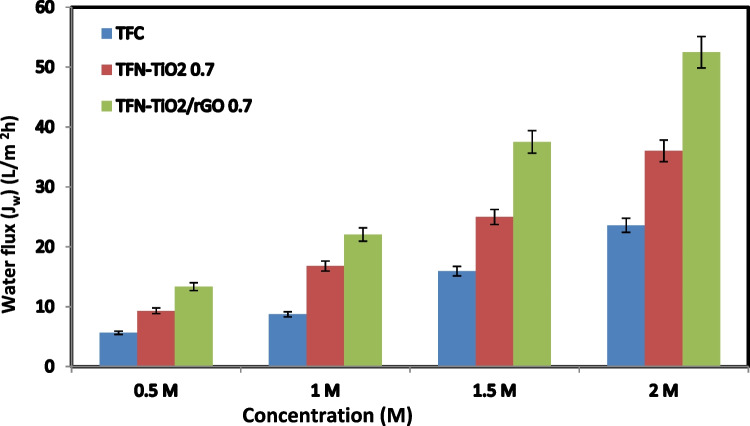
Water flux at different concentrations of NaCl (0.5, 1, 1.5, 2 M) as a draw solution and 0.1 M of NaCl as a feed solution

The efficiency of thin-film hydride composite (TFC) and thin-film hydride nanocomposite (TFN-TiO_2_) skin membranes, in addition to thin-film hydride nanocomposite containing reduced graphene oxide (TFN-TiO_2_/rGO), might be affected by the feeding and drawing ratios employed during the membrane fabrication technique. The water flux for TFC, TFN-TiO_2_, and TFN-TiO_2_/rGO 0.5 membranes at varied DS concentrations is shown in Fig. 7 (0.5, 1, 1.5, and 2 M NaCl). It should also be highlighted that the TFC and TFN membranes show the same development pattern for both the water and reverse salt flux, with rising extraction solution concentrations within the FO system. The extraction solution’s increased osmotic driving force makes the water flow climb steadily as concentration rises. Contrarily, the TFN-TiO_2_/rGO 0.5 membranes consistently showed a substantially greater water flux calculated from the increased water permeability, regardless of the extraction solution’s concentration or the membrane’s orientation (Niksefat et al. 2014; Shen et al. 2016; Xu et al. 2019; Li et al. 2021).

**Fig. 8:**
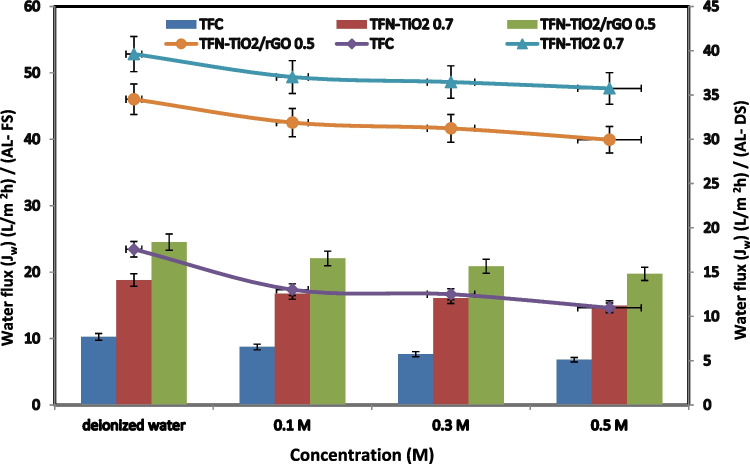
Water flux at different concentrations of NaCl (deionized water, 0.1, 0.3, 0.5 M) as a feed solution and 1 M of NaCl as a draw solution in both orientations (AL-FS and AL-DS)

Even when there is a short-term increase in water flow, the increase in total water drops when the ICP technique in the membrane supporting layer becomes more dilute, along with increases in concentration. As the DS increases in concentration, the water flowing serves as a limiting component, lowering the DS’s efficiency. This demonstrates that regardless of how much osmotic pressure DS can generate, it cannot provide the same amount of water permeability as the FO approach (Pang et al. 2019; Singh et al. 2021a). Figure 8 shows a study of FO water performance using 0.7 TNF-TiO_2_ and 0.5 TNF-TiO_2_/rGO membranes at different feed concentrations. At different feed concentrations (0–0.5 M NaCl) and constant 1 M NaCl as DS, the AL-DS and AL-FS orientations of the FO membranes were also examined. When the FS dosage level exceeded 0.1 M NaCl, the water flow across both FO membranes with the AL-DS orientation progressively declined, but abruptly when it did. On top of that, the orientation of AL-FS resulted in a steady decrease in water flux as feed concentration increased. For the entire range of FS concentrations considered, TFN-TiO_2_ 0.7 and TFN-TiO_2_/rGO 0.5 membranes outperformed the TFC membrane in terms of FO water flow despite the clear profile variations between the two orientations. The TFN membrane may be more favorable to be employed when processing feeding solutions with somewhat salinity-higher water under the AL-FS orientation (Darabi et al. 2018; Al-Najar et al. 2020).

## Conclusion

In situ interfacial polymerization technique was employed for the successful fabrication of thin-film nanocomposite (TFN) membranes containing TiO_2_ in addition to TiO_2_/rGO nanocomposites. The fabrication of polyamide-TiO_2_ and TiO_2_/rGO nanocomposite membranes was verified using FT-IR, AFM, and SEM spectroscopies. After the incorporation of TiO_2_ together with TiO_2_/rGO nanoparticles, cellular membranes’ hydrophilicity along with porosity was improved. The thin-film nanocomposite membranes’ reverse solute flux and water flux were also enhanced. The TFN-FO membrane with 0.5 wt% TiO_2_/rGO and the membrane with 0.7 wt% TiO_2_ have water flux and reverse solute flux (24.52 and 18.81 L m^−2^h^−1^ and 2.74 and 2.15 g/m^2^ h, respectively), while for TFC, the water flux and reverse solute flux (10.24 L m^−2^h^−1^ and 6.53 g/m^2^ h). The study shows that modifying TFC membranes with TiO_2_/rGO can effectively improve forward osmosis water flux. In summary, the key findings are that TiO_2_/rGO nanocomposite enhances the hydrophilicity and porosity and compared the water flow through TFC membranes with TiO_2_ alone. Among the manufactured membranes, the TFC-FO membrane of 0.5 wt% TiO_2_/rGO exhibited the maximum water flow among the synthesized membranes.
